# Supplementary material for: The Unknown Health Burden of Herpes Zoster Hospitalizations: The Effect on Chronic Disease Course in Adult Patients ≥50 Years
Source: Vaccines (Basel). 2020 Jan 10;8(1):20. doi: 10.3390/vaccines8010020 (PMC7157675; doi:10.3390/vaccines8010020)
Supplement: Supplementary file 1 [file vaccines-08-00020-s001.pdf]

Table S1. Further hospitalizations during the 6 months after hospitalization in HZ cohort and non-HZ cohort, period 2015-2017.

Major Diagnostic Category	HZ cohort (%, 95%CI)	Non-HZ cohort (%, 95%CI)	Relative risk RR (95% CI)	p value
**01 - Diseases and Disorders of the Nervous System**	5.56 (3.25; 7.86)	4.24 (3.32; 5.16)	1.31 (0.82; 2.10)	0.281
**02 - Diseases and Disorders of the Eye**	1.59 (0.33; 2.85)	0.54 (0.21; 0.88)	2.92 (1.07; 7.99)	0.031
**03 - Diseases and Disorders of the Ear, Nose, Mouth And Throat**	0.00 (0.0; 0.0)	0.16 (-0.02; 0.35)	-	0.433
**04 - Diseases and Disorders of the Respiratory System**	6.08 (3.67; 8.49)	4.35 (3.41; 5.28)	1.40 (0.89; 2.20)	0.165
**05 - Diseases and Disorders of the Circulatory System**	3.97 (2.00; 5.94)	7.28 (6.09; 8.47)	0.55 (0.32; 0.92)	0.027
**06 - Diseases and Disorders of the Digestive System**	4.23 (2.20; 6.26)	4.67 (3.71; 5.64)	0.91 (0.54; 1.53)	0.723
**07 - Diseases and Disorders of the Hepatobiliary System And Pancreas**	1.59 (0.33; 2.85)	2.12 (1.46; 2.78)	0.75 (0.32; 1.76)	0.512
**08 - Diseases and Disorders of the Musculoskeletal System And Connective Tissue**	4.50 (2.41; 6.59)	6.63 (5.49; 7.76)	0.68 (0.41; 1.11)	0.141
**09 - Diseases and Disorders of the Skin, Subcutaneous Tissue And Breast**	1.32 (0.17; 2.47)	1.47 (0.92; 2.02)	0.90 (0.35; 2.33)	0.833
**10 - Diseases and Disorders of the Endocrine, Nutritional And Metabolic System**	0.53 (-0.20; 1.26)	0.49 (0.17; 0.81)	1.08 (0.24; 4.99)	0.919
**11 - Diseases and Disorders of the Kidney And Urinary Tract**	3.17 (1.41; 4.94)	4.24 (3.32; 5.16)	0.75 (0.41; 1.36)	0.358
**12 - Diseases and Disorders of the Male Reproductive System**	0.26 (-0.25; 0.78)	0.38 (0.10; 0.66)	0.70 (0.09; 5.64)	0.733
**13 - Diseases and Disorders of the Female Reproductive System**	0.00 (0.0; 0.0)	0.38 (0.10; 0.66)	-	0.231
**14 - Pregnancy, Childbirth And Puerperium**	0.00 (0.0; 0.0)	0.00 (0.0; 0.0)	-	-
**15 - Newborn And Other Neonates (Perinatal Period)**	0.00 (0.0; 0.0)	0.00 (0.0; 0.0)	-	-
**16 - Diseases and Disorders of the Blood and Blood Forming Organs and Immunological Disorders**	1.85 (0.49; 3.21)	1.58 (1.01; 2.14)	1.18 (0.52; 2.66)	0.703
**17 - Myeloproliferative Disorders (Poorly Differentiated Neoplasms)**	4.76 (2.62; 6.91)	6.63 (5.49; 7.76)	0.72 (0.44; 1.16)	0.199
**18 - Infectious and Parasitic Disorders (Systemic or unspecified sites)**	3.97 (2.00; 5.94)	2.01 (1.37; 2.65)	1.97 (1.09; 3.56)	0.026
**19 - Mental Diseases and Disorders**	0.00 (0.0; 0.0)	0.43 (0.13; 0.74)	-	0.200
**20 - Alcohol/Drug Use or Induced Mental Disorders**	0.00 (0.0; 0.0)	0.05 (-0.05; 0.16)	-	0.650
**21 - Injuries, Poison And Toxic Effect of Drugs**	0.79 (-0.10; 1.69)	0.33 (0.07; 0.59)	2.44 (0.61; 9.69)	0.195
**22 - Burns**	0.00 (0.0; 0.0)	0.00 (0.0; 0.0)	-	-
**23 - Factors Influencing Health Status and Other Contacts with Health Services**	1.06 (0.03; 2.09)	1.58 (1.01; 2.14)	0.67 (0.24; 1.90)	0.455
**24 - Multiple Significant Trauma**	0.00 (0.0; 0.0)	0.11 (-0.04; 0.26)	-	0.522
**25 - Human Immunodeficiency Virus Infection**	1.06 (0.03; 2.09)	0.00 (0.0; 0.0)	-	<0.001
**MDC Category Missing**	0.00 (0.0; 0.0)	0.33 (0.07; 0.59)	-	0.267
**Pre-MDC**	0.00 (0.0; 0.0)	0.16 (-0.02; 0.35)	-	0.433

Table S2. Days of drug therapy in the 6-months after and before hospitalization in HZ cohort and non-HZ cohort, period 2015-2017.

ATC codes	Pre-H HZ cohort (median, IQR)	Pre-H non-HZ cohort (median, IQR)	p value pre-H	Post-H HZ cohort (median, IQR)	Post-H non-HZ cohort (median, IQR)	p value post-H	p value post- vs pre-H in HZ and non-HZ
**A-Alimentary tract and metabolism**	168 (67.5-280.75)	146 (68.63-262.21)	0.078	153 (63-270)	152 (70-275)	0.901	0.294
**B-Blood and blood forming organs**	140 (60-202.3)	150 (79-220)	0.878	134 (60-222)	150 (60-255)	0.179	0.026
**C-Cardiovascular system**	260 (126.3-504)	285 (150-504)	0.279	243 (120-457)	292 (144-504)	0.003	0.046
**D-Dermatologicals**	40 (20-60)	33 (30-60)	0.686	23 (3-62)	50 (30-95)	0.171	0.278
**G-Genito-urinary system and sex hormones**	160 (112-220)	160 (100-240)	0.639	154 (60-210)	180 (100-240)	0.152	0.281
**H-Systemic hormonal preparations excluding sex hormones and insulins**	67.67 (26.7-139.6)	64 (30-125)	0.488	67 (30-133)	60 (25-125)	0.532	0.748
**J-Anti-infectives for systemic use**	12.5 (7-24)	10.5 (6-22.5)	0.403	15 (7-29)	12 (6-22)	0.001	0.236
**J05AB11-Valaciclovir**	7 (7-11)	11 (7-23)	0.035	7 (7-7)	14 (7-49)	0.099	<0.001
**J05AB09-Famciclovir**	14 (7-18)	11 (7-14)	0.553	7 (7-7)	14 (14-14)	0.317	0.005
**J05AB01-Aciclovir**	7 (6-7)	7 (5-14)	0.423	7 (7-7)	7 (5-14)	0.876	0.029
**J05AB15-Brivudine**	7 (7-7)	7 (7-7)	0.263	7 (7-9)	7 (7-7)	0.507	<0.001
**L-Antineoplastic and immunomodulating agents**	29 (6-115)	72.24 (16.7-180)	0.006	59 (25-163)	90 (24-180)	0.158	0.004
**M-Musculo-skeletal system**	40 (20-83)	44 (20-90)	0.439	36 (20-83)	45 (20-90)	0.270	0.115
**N-Nervous system**	77 (20-168)	72.4 (21.3-180)	0.712	83.08 (22.31-181.06)	72 (18-180)	0.477	<0.001
**N03AX16-Pregabalin**	14 (4-39)	28 (11-63)	0.002	25 (8-49)	18 (9-44)	0.684	<0.001
**N03AX12-Gabapentin**	17 (8-66)	26 (8-49)	0.338	25 (8-44)	25 (8-42)	0.921	<0.001
**N06AA09-Amitriptyline**	11 (11-43)	21.34 (11-43)	0.298	21 (11-43)	21 (11-64)	0.654	0.00
**N01BB02-Lidocaine**	7 (4-18)	7 (4-12)	0.907	9 (4-22)	4 (4-7)	0.071	<0.001
**N02BE-Anilides**	11 (5-21)	11 (5-21)	0.823	11 (11-21)	11 (5-21)	0.414	0.582
**N02A-Opioids**	11 (5-46)	13 (5-43)	0.301	16 (6-52)	11 (5-38)	<0.001	0.003
**P-Antiparasitic products. insecticides and repellents**	7 (3-12)	5 (3-47)	0.957	12 (5-47)	5 (3-13.32)	0.198	0.353
**R-Respiratory system**	60 (30-180)	60 (30-176)	0.855	60 (30-180)	63 (30-180)	0.906	0.176
**S-Sensory organs**	150 (44-250)	175 (100-275)	0.694	75 (33-225)	150 (100-250)	0.051	0.968
**V-Various**	0 (0-10)	0 (0-19)	0.640	1 (0-9)	0 (0-12)	0.524	0.842
**Not specified**	0 (0-0)	0 (0-0)	-	0 (0-0)	0 (0-0)	-	1.000
